# Cross-Talk Between the Tumor Microenvironment, Extracellular Matrix, and Cell Metabolism in Cancer

**DOI:** 10.3389/fonc.2020.00239

**Published:** 2020-02-26

**Authors:** Mona Nazemi, Elena Rainero

**Affiliations:** Biomedical Science Department, The University of Sheffield, Sheffield, United Kingdom

**Keywords:** extracellular matrix, cell metabolism, cancer associated fibroblasts, nutrient scavenging, nutrient signaling

## Abstract

The extracellular matrix (ECM) is a complex network of secreted proteins which provides support for tissues and organs. Additionally, the ECM controls a plethora of cell functions, including cell polarity, migration, proliferation, and oncogenic transformation. One of the hallmarks of cancer is altered cell metabolism, which is currently being exploited to develop anti-cancer therapies. Several pieces of evidence indicate that the tumor microenvironment and the ECM impinge on tumor cell metabolism. Therefore, it is essential to understand the contribution of the complex 3D microenvironment in controlling metabolic plasticity and responsiveness to therapies targeting cell metabolism. In this mini-review, we will describe how the tumor microenvironment and cancer-associated fibroblasts dictate cancer cell metabolism, resulting in increased tumor progression. Moreover, we will define the cross-talk between nutrient signaling and the trafficking of the ECM receptors of the integrin family. Finally, we will present recent data highlighting the contribution of nutrient scavenging from the microenvironment to support cancer cells growth under nutrient starvation conditions.

## Introduction

Cancer-associated fibroblasts (CAFs) are a heterogenous and plastic population of activated fibroblasts, which represent a significant proportion of the tumor microenvironment. For instance, CAFs can account up to 70 and 90% of breast and pancreatic cancer tumor mass, respectively (1, 2). The role of CAFs in tumorigenesis is widely established and they have been shown to contribute to tumor growth, metastasis and resistance to therapy (3). Moreover, CAFs play an important role in the regulation of cancer metabolism, primarily through the secretion of metabolites and the generation of a stiffer and fibrotic ECM, which in turn affects cancer cell metabolism.

Cells interact with the ECM through plasma membrane receptors. Integrins are transmembrane receptors that regulate cell adhesion, migration, and mechanotransduction through mediating cell-ECM interaction (4, 5). Integrins can trigger different intracellular signaling pathways promoting cell growth, survival, and proliferation (6). Furthermore, ligand bound-integrin trafficking has recently been shown to directly or indirectly affect nutrient signaling (7, 8). Mechanistic target of Rapamycin (mTOR) signaling pathway is the key regulator of anabolic and catabolic processes of the cells. During nutrient availability, mTOR induces anabolic processes such as protein, nucleotide, and lipid biosynthesis and inhibits cellular autophagy and lysosomal biogenesis (9–11). mTOR forms two independent complexes, mTORC1 and mTORC2. mTORC1 adjusts cell growth and proliferation in response to growth factors and amino acids, while mTORC2 has a role in actin organization. Furthermore, mTORC2 can control cell proliferation and survival through AKT activation downstream of growth factor signaling (12). During nutrient starvation, the activity of mTORC1 is restrained, allowing the cells use other sources of nutrient acquisition, such as autophagy (13).

In cancer cells, the activation of oncogenic signaling has a profound effect on cell metabolism. Indeed, aberrant activation of phosphatidyl inositol 3 kinase (PI3K)/AKT and Ras signaling pathways facilitates constant glucose uptake through GLUT1 receptor (14, 15). In parallel, derailed function of the oncogenic protein c-myc induces the expression of glutamine transporters and glutamine-utilizing enzymes (16, 17). In addition, high rate of tumor growth and insufficient vasculature forming deep inside the tumors significantly increase the chance of nutrient scarcity in the tumor microenvironment (TME) (18). Indeed, the TME has been shown to be depleted of amino acids and glucose (19, 20).

In this mini-review, we will highlight how CAFs control cancer cell metabolism, through metabolite secretion and the generation of a stiffer microenvironment. We will then summarize the cross-talk between integrin trafficking, metabolism and nutrient signaling and we will describe the different mechanisms through which cancer cells can exploit unconventional nutrient sources.

## The Tumor Microenvironment and CAFs Dictate Changes in Cancer Cell Metabolism

The activation of fibroblasts to CAFs is coupled with a metabolic shift to glycolysis, increased catabolic activity and autophagy (21). As a result of this, CAFs secrete a variety of metabolites that can support cancer cell growth and metabolism.

**Figure 1:**
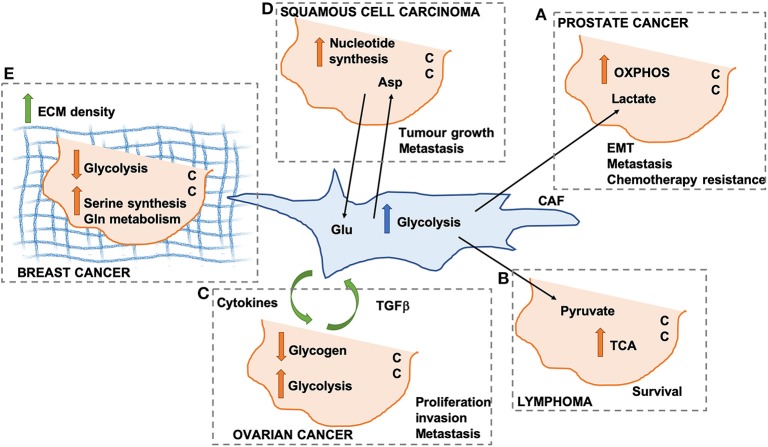
Schematic representation of different mechanisms through which cancer-associated fibroblasts (CAFs) can modify cancer cell (CC) metabolism. OXPHOS, oxidative phosphorylation; TCA, tricarboxylic acid cycle; Glu, glutamate; Asp, aspartate; ECM, extracellular matrix. **(A)** Lactate secreted by CAFs promotes OXPHOS in pancreatic CCs. **(B)** CAF-derived pyruvate promotes TCA activation in lymphoma CCs. **(C)** Cytokines and TGF beta promote CAF-ovarian CC cross-talk. **(D)** Asp secreted by CAFs drives nucleotide synthesis in squamous cell carcinoma. **(E)** ECM density promotes Serine synthesis and Gln metabolism in breast cancer.

In prostate cancer, CAFs promote the so-called reverse Warburg effect, whereby lactate secretion and mitochondria transfer from CAFs to cancer cells (CCs) lead to an increase in mitochondrial activity and oxidative phosphorylation (OXPHOS) in CCs (Figure 1A). This promotes epithelial-to-mesenchymal transition, metastatic burden, and chemotherapy resistance (22). Similarly, the secretion of pyruvate by CAFs has been shown to promote lymphoma cell survival through the upregulation of the tricarboxylic acid (TCA) cycle (23) (Figure 1B), as well as ECM remodeling and lung metastasis in breast cancer, through the upregulation of alpha ketoglutarate production (24).

In ovarian cancer, CAFs have been shown to promote glycogen metabolism and glycolysis in CCs, through the production of cytokines, which is in turn promoted by CC-derived TGFβ. This CAFs-CC signaling loop results in increase CC proliferation, invasion and metastasis (25) (Figure 1C). It is important to note that all the afore-mentioned studies were performed using conditioned media from CAFs or short-term co-culture experiments (a few hours). Despite providing useful insight in the contribution of secreted factors, these fail to recapitulate the complex 3D structure of the TME, whereby changes in ECM composition and properties could play a role in the metabolic reprogramming observed in these studies. Indeed, the stiffness of the TME has been shown to have profound effects on both CAF and CC metabolism in squamous cell carcinoma, leading to an increase in glycolysis and glutamine consumption in both cell populations. In particular, the activation of the YAP/TAZ signaling pathway in stiffer environments promotes the expression of glycolytic enzymes (26), as well as glutamine uptake and conversion to glutamate in CCs. Glutamate is secreted by CCs and utilized by CAFs for maintaining redox homeostasis through the glutathione pathway. CAFs, on the other hand, secrete aspartate, which is used by CCs to promote nucleotide synthesis (27) (Figure 1D). Similar results have been observed *in vivo* in an orthotopic mouse model of breast cancer, where ECM stiffness is coupled with increased glycolysis, glutamine metabolism and aspartate production. Importantly, a reduction in intratumoral levels of aspartate and glutamine leads to a decrease in cell proliferation (27). Interestingly, glutamate secretion by CCs is also involved in the invasive phenotype of breast cancer cells. Indeed, by binding to its receptor GRM3, glutamate promotes the trafficking of the matrix metalloprotease MT1-MMP, leading to CC invasive migration (28).

CAFs have a pivotal role in determining the composition and organization of the ECM within the TME, although other cell types have been shown to contribute to ECM secretion and deposition. Increased collagen density is observed during carcinoma progression and has been shown to be mainly produced by CAFs (29). Importantly, changes in collagen density have been linked with alterations in breast cancer cell metabolism. High density collagen gels are associated with a reduction of oxygen consumption and the amount of glucose that is metabolized through the TCA cycle, with a concomitant increase in the use of glutamine to fuel the TCA cycle. This is associated with a reduction in the expression of glycolysis genes and an increase in oxidative glutamine metabolism, serine synthesis and one carbon metabolism enzymes (30) (Figure 1E). This is in contrast with the data presented above by Bertero et al. (27). This could be due to differences in the type of cells analyzed (ovarian cancer vs. breast cancer), or the experimental settings (hydrogels with different stiffnesses vs. collagen I gels with different densities). Moreover, independent sets of metabolic enzymes where shown to be modulated in the two studies, suggesting that separate mechanotransduction pathways could impinge on cell metabolism. Within the TME, the ECM is constantly remodeled through the action of matrix degrading enzymes. Recent data indicate that the degradation of hyaluronan, a ubiquitous ECM component, promotes glucose uptake in several cancer cell lines, by increasing the plasma membrane localization of the glucose transporter GLUT1. This in turns fosters glycolysis and promotes cancer cell migration (31). It is unclear whether the degradation of other ECM components is also linked to changes in cell metabolism. Moreover, future work will be needed to characterize how ECM degradation in more complex 3D environments impinges on CC metabolism.

## The Crosstalk Between Cell Metabolism and the Trafficking and Function of the ECM Receptors of the Integrin Family

**Figure 2:**
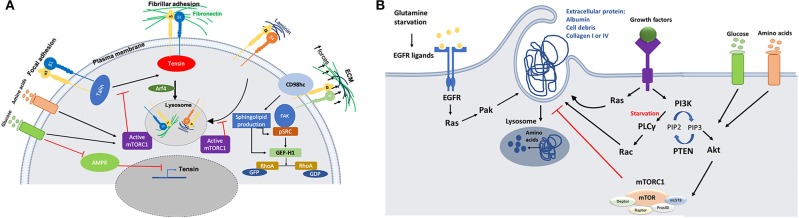
**(A)** Schematic representation of the crosstalk between integrins trafficking and cell metabolism. Nutrient starvation induces ligand (fibronectin or Laminin)-bound integrin internalization through mTORC1 deactivation. On the other hand, activation of AMPK during starvation inhibits tensin expression, opposing integrin activation. There is also a link between integrin mechanosensing and sphingolipid production through CD98hc protein, which is an integrin co-receptor sensing mechanical forces. Activation of CD98hc induces sphingolipid production leading to activation of Src and GEF-H1 upstream of RhoA. **(B)** Schematic representation of the molecular mechanisms through which nutrient starvation controls macropinocytosis. Macromolecules internalization under nutrient starvation is induced through Ras and growth factor dependent mechanisms. Amino acids extracted from protein uptake can be used to feed the TCA cycle to make ATP or they may be used to make other proteins.

mTOR is one of the signaling pathways that has been shown to be regulated by integrin trafficking. It has been demonstrated that in ovarian cancer cells, glucose starvation induces the translocation of α5β1 integrin from peripheral focal adhesions to a centrally located patch of fibrillar adhesions. This has been identified as the main internalization site for fibronectin (FN)-bound α5β1, resulting in tensin and Arf4-dependent endocytosis and lysosomal delivery of FN-bound α5β1. This internalization pathway leads to mTORC1 lysosomal recruitment and activation (Figure 2A). Mimicking nutrient depleted environments by inhibiting mTORC1 activity leads to higher ligand-bound α5β1 integrin endocytosis and tensin-dependent sub-nuclear adhesion formation, showing that tensin-dependent α5β1 internalization is controlled by nutrient availability (7). Serum and growth factor starvation also induce normal mammary epithelial cells to internalize soluble laminin through β4 integrin; this mediates an increase in the cellular amino acid content through laminin lysosomal degradation and amino acid extraction, which in turn returns mTORC1 activity to its normal condition to avoid excess uptake of extracellular proteins (Figure 2A). *In vivo* studies also reveal that mammary epithelial cells in dietary restricted mice increase laminin uptake from the basement membrane and fibroblast ECM secretion (8). Altogether, these data indicate that the uptake of ECM components, regulated by integrin trafficking under nutrient deficient conditions, could provide a source of nutrients for cancer cells. However, more studies are required to investigate whether different integrin heterodimers are involved in the internalization of other ECM components and whether this is required for nutrient signaling as well.

AMP-activated protein kinase (AMPK) is a serine/threonine kinase that also works as a sensor for the availability of nutrients. Lack of energy and cell exposure to certain levels of stress activate AMPK, which inhibits anabolic pathways and induces catabolic pathways to provide more energy or source of nutrition for the cells (32). It has been revealed that AMPK can inhibit the activation of α5β1 integrins in fibroblasts. In particular, AMPK reduces tensin3 expression, thus preventing integrin activation (Figure 2A). In AMPK KO mouse embryonic fibroblasts (MEFs), the activity of α5β1 is increased, leading to more fibrillar adhesion formation and fibronectin biogenesis (33). More evidence is required to evaluate the effect of higher fibronectin biogenesis on nutrient signaling/availability for cells, since previous studies highlighted that active integrins in fibrillar adhesion would lead to higher fibronectin endocytosis under starvation (7). In addition, since cancer cells are more prone to face nutrient scarcity, more investigations are required to elucidate the effect of AMPK activity in those cells. Even though studies on mTORC1 activity suggest that nutrient deficiency assist cells to benefit from alternative food sources such as fibronectin and laminin through receptor dependent (integrin)-endocytosis, this study shows that starvation could also have an inhibitory effect on integrin membrane transport and trafficking. As it has been reviewed by Georgiadou and Ivaska (34), AMPK activity in cancer cells and cancer associated fibroblast cells (CAFs) lead to a cross-talk between them. Low activity of AMPK in CAFs induces tensin expression leading to upregulation of ECM secretion and formation of fibrillar adhesion. In contrast, high AMPK activity in cancer cells under starvation inhibits mTORC1 activity which results in ECM internalization and lysosomal degradation to provide nutrient for the cells.

In addition to signaling pathways that directly deal with nutrient availability around the cells, a link between integrin mechanosensing and cell metabolism has also been proposed. CD98hc is an integrin co-receptor and amino acid transporter sensing mechanical forces and causing cell stiffening through integrin and RhoA activation. It has been demonstrated that there is a cross-talk between mechanosensing interruption and sphingolipid synthesis in fibroblasts bearing C330S mutation in CD98hc. Sphingolipids are essential membrane components that can regulate transmembrane protein dynamics. Loss of CD98hc reduces sphingolipid availability, leading to defects in movement, recruitment and activation of regulatory proteins upstream of RhoA (Figure 2A). Therefore, it is suggested that there is a cross-talk between sensing the mechanical forces that cells are exposed to and their metabolic state. This suggests that cancer cells could benefit from different environmental mechanical forces through metabolic adaptation. Indeed, it has been demonstrated that circulating tumor cells display an oxidative switch, while the cells in the primary tumor are mostly glycolytic (35). An intriguing explanation for this phenotype could be the loss of mechanical forces present in the primary tumor. Further studies are needed to address this point, as invasive cancer cells experience different mechanical forces during their migration (36).

## Strategies Undertaken by Cancer Cells to Scavenge Nutrients From the Microenvironment

Even though blood pressure inside the tumors is low due to high interstitial pressure, lymphatic deficiency and leaky blood vessels increase the accessibility of cancer cells to the blood serum or its main protein, albumin (37, 38). Ras-driven cancer cells have a higher rate of macropinocytosis which helps them to internalize extracellular proteins (ECPs). In Ras-driven pancreatic ductal adenocarcinoma cells (PDACs) and Ras-transformed MEFs, amino acid starvation induces albumin macropinocytosis followed by lysosomal degradation and amino acid extraction (Figure 2B). It has also been revealed that mimicking nutrient starvation by inhibiting mTORC1 signaling pathway induces cells to rely on extracellular macromolecules rather than amino acids (19, 39, 40). Lack of nutrient and oxygen delivery leads to cell death at the center of tumors. In PTEN-deficient prostate cancer cell lines and KRas-driven pancreatic cancer cells, AMPK activation and mTORC1 suppression under amino acid and glucose starvation assist cell proliferation through inducing cell debris scavenging. It was revealed that amino acids extracted from cell debris can participate in building cell biomass (41). In addition to the access to albumin and cell debris, PDAC cells are also surrounded by a dense network of ECM containing collagen I and collagen IV. PDAC cells are able to internalize collagen I and IV through macropinocytosis under glucose starvation and receptor-dependent endocytosis under low glutamine conditions (Figure 2B). PDAC cells degrade the internalized collagens in the lysosomes, providing a source of proline. Proline is then fed into the TCA cycle, leading to ATP production and cell survival, via the activation of the ERK1/2 pathway. Contrary to albumin, collagen uptake in this context does not affect mTORC1 signaling pathway, and the role of mTOR inhibition has not been addressed (42). It is not clear whether other amino acids from the collagen also contribute to cancer cell survival. Even though the role of Ras has been highlighted in terms of inducing macropinocytosis under starvation conditions, its role could be dispensable in the presence of growth factors. Indeed, it has been demonstrated that MEFs are be able to induce ECP uptake through growth-factor (GF) dependent macropinocytosis, in a PI3 kinase-dependent manner (Figure 2B). Under amino acid starvation, activation of Rac1 and PLCγ as an PI3K effector induces ECP macropinocytosis, while availability of glucose and amino acids induces AKT-dependent activation of mTORC1 signaling. This allows cells to use free amino acid from transporters instead of macropinocytosis to uptake macromolecules (43). PDAC cells are heterogenous in terms of macropinocytosis potential. A subset of PDAC tumors have been shown to upregulate macropinocytosis under glutamine starvation conditions, while other tumors display constitutive macropinocytosis which is not affected by the level of nutrients. Mechanistically, glutamine starvation potentiates EGFR signaling, leading to the activation of the Ras/Pak signaling pathway. This in turn induces ECP macropinocytosis, followed by lysosomal degradation (44). Along with *in vitro* studies showing that cancer cells can use albumin as an alternative source of nutrients, hypoalbuminemia was also seen in some cancer patients (45). An *in vivo* study directly showed that PDAC cells inside the tumor are able to internalize albumin through macropinocytosis and use albumin derived amino acids for further metabolic pathways, while adjacent normal cells do not have this ability (46). In addition to taking advantage of unusual protein sources, cancer cells are able to use extracellular lipids for their survival under hypoxia or starvation conditions. Cancer cells need lipid and fatty acids to reproduce their membrane and proliferate. In normal oxygen conditions (normoxia), cancer cells can produce non-essential fatty acids. In contrast, hypoxic or Ras- driven cancer cells rely on scavenging fatty acids from TME. Ras-driven cancer cells can internalize serum lipids with one fatty acid tail (lysophospholipid) to compensate the lack of lipid production (47). Under glucose and amino acid starvation lipid droplet (LP) content of cancer cells declines. PTEN-deficient prostate cancer cells rely on lipids extracted from cell debris through macropinocytosis to replenish their LP storage containing fatty acids and cholesterol (41). Invasive breast cancer cells also show higher proliferation and migration in co-culture with obese adipocytes. Transferring of fatty acids from adipocytes to invasive breast cancer cells induces adipose triglyceride lipase (ATGL)-mediated lipolysis and oxidation of fatty acids in mitochondria (48).

Since cancer cells could face different nutrient conditions in various niches during their development, further work is needed to elucidate how ECP and ECM could affect nutrient availability and how Ras and PI3K pathways would interact and affect each other in response to different nutrient conditions.

## Conclusions and Future Directions

Cancer cells display an elevated metabolic plasticity, allowing them to adapt to different environments and nutrient levels. The TME, in particular CAFs and cell-ECM interaction, are key in controlling this metabolic switch. CAFs secrete several metabolites, including lactate, pyruvate and aspartate, which have been shown to support the growth of different cancer cell types (3). Moreover, through the generation of a stiffer ECM, CAFs drive changes in cancer cell metabolism, leading to an increase in one-carbon metabolism and oxidative glutamine metabolism in the TCA cycle. Furthermore, ECM components, such as fibronectin and laminin, have been shown to be internalized in an integrin-dependent manner and to control nutrient signaling. On the other hand, it is now clear that nutrient-dependent signaling pathways can control integrin trafficking and activation in diverse ways. However, more work needs to be done to elucidate how nutrient starvation impinges on vesicular trafficking and receptor internalization in complex 3D environments.

Elevated rates of tumor growth and limited blood supply result in a nutrient-deprived TME. These pushes cancer cells to adopt different strategies to obtain metabolic fuels. These include the internalization of ECP, ECM and cell debris. Despite all these processes have been observed, the cross-talk between different nutrient scavenging strategies and how cells can switch between them have not been fully addressed yet. Importantly, it is not known how changes in the TME, including immune cell infiltration and ECM composition and structure, affect the ability of cancer cells to grow under nutrient deprived conditions.

Altogether, understanding how cancer cells can thrive within the challenging TME will lead to the identification of cancer vulnerabilities. These could then be exploited for the development of novel strategies for targeting unresponsive tumors.

## Author Contributions

MN and ER wrote this manuscript and generated the figures.

### Conflict of Interest

The authors declare that the research was conducted in the absence of any commercial or financial relationships that could be construed as a potential conflict of interest.
